# MRI efficacy in diagnosing internal lesions of the knee: a retrospective analysis

**DOI:** 10.1186/1752-2897-2-4

**Published:** 2008-06-02

**Authors:** Vassilios S Nikolaou, Efstathios Chronopoulos, Christianna Savvidou, Spyros Plessas, Peter Giannoudis, Nicolas Efstathopoulos, Georgios Papachristou

**Affiliations:** 1 Academic Department of Trauma & Orthopaedics, Leeds Teaching Hospitals, School of Medicine, University of Leeds, UK; 2 2nd Academic Department of Trauma & Orthopaedics, Konstantopoulion Hospital, Athens University, Greece

## Abstract

**Background:**

Many surgeons tend to believe that MRI is an accurate, non invasive diagnostic method, enough to lead to decisions for conservative treatment and save a patient from unnecessary arthroscopy. We conducted a retrospective study to investigate the accuracy of the MRI of the knee for the detection of injuries of the meniscus, cruciate ligaments and articular cartilage, in comparison with the preoperative clinical examination and intraoperative findings. Between May 2005 and February 2006 102 patients after clinical examination were diagnosed with meniscal or cruciate injury and underwent definitive treatment with arthroscopy. 46 of these patients fulfilled the inclusion criteria. The accuracy, sensitivity, specificity, negative and positive predictive values of the MRI findings were correlated with the lesions identified during arthroscopy. The diagnostic performance of the initial clinical examination was also calculated for the meniscal and cruciate ligament injuries.

**Results:**

The accuracy for tears of the medial, lateral meniscus, anterior and posterior cruciate ligaments and articular cartilage was 81%, 77%, 86%, 98% and 60% respectively. The specificity was 69%, 88%, 89%, 98% and 73% respectively. The positive predictive value was 83%, 81%, 90%, 75% and 53% respectively. Finally, the clinical examination had significant lower reliability in the detection of these injuries.

**Conclusion:**

MRI is very helpful in diagnosing meniscal and cruciate ligament injuries. But in a countable percentage reports with false results and in chondral defects its importance is still vague. The arthroscopy still remains the gold standard for definitive diagnosis.

## Background

Arthroscopy is considered as "the gold standard" for diagnosis of traumatic intraarticular knee lesions [1]. However, arthroscopy is an invasive procedure that requires hospitalization and anaesthesia, thus presenting all the potential complications of a surgical procedure [2]. Since it's introduction in the 1980's Magnetic Resonance Imaging (MRI) has gained in popularity as a diagnostic tool of the musculoskeletal disorders [3]. Especially the knee is the most frequent examined joint with MRI. Many surgeons tend to believe that MRI is an accurate, non invasive diagnostic method of the knee injuries, enough to lead to decisions for conservative treatment and save a patient from unnecessary arthroscopy. Nevertheless, even nowadays, remains very expensive. Taking in account that health-economics play important role in patients management, many questions arise regarding when and how often one must ask for an MRI when clinical examination has already confirm the diagnosis of meniscal tear or cruciate ligament rupture [4]. The opposite question might be more important; is negative MRI enough to prevent unnecessary arthroscopy, when clinical examination suggests a meniscal or cruciate ligament injury?

With the purpose of investigating the accuracy of magnetic resonance imaging in patients with clinical signs of traumatic intraarticular knee lesions, we compared its findings with those obtained from the subsequent arthroscopies.

## Methods

After obtaining the approval of the hospital ethics committee, we retrospectively reviewed the case notes of patients who had been clinically diagnosed with meniscal or cruciate injury, between May 2005 and February 2006 in our institution. Patients who had subsequently undergone further examination with MRI and were definitively treated with arthroscopy were then identified. We adhered to the Standards for Reporting of Diagnostic Accuracy (STARD) criteria for design and presentation of diagnostic studies [5]

Patients that in plain X-rays had fractures, loose bodies or signs of severe osteoarthritis were excluded from the study. Additionally, patients that after the MRI examination have had new injury to the same knee, before the arthroscopy or delayed to undergo arthroscopy for more than 3 months, were also excluded.

All patients had thorough clinical examination from two experienced knee surgeons prior to the MRI. Clinical examination focused on meniscal injury and cruciate ligament injury. The tests used in the clinical diagnosis were: the anterior-posterior drawer test, the Lachman test, the pivot shift test for the diagnosis of cruciate ligament injuries and the Apley's and McMurray's test for the meniscal injuries [6].

MRI examinations were performed in 2 different diagnostic centres. The MRI scanners were two 1.5 tesla units (Philips Medical Systems). T1 and T2 weighted images in coronal, axial and sagittal planes were obtained. Slice thickness ranged from 3 to 5 mm. The films were interpreted from 2 experienced knee radiologists who were aware of the result of the clinical examination as this was written at the initial referral letter. Any abnormalities of the cruciate ligaments, menisci or hyaline cartilage were described on a standard form. Preoperatively each MRI was also assessed by the surgeon performing the arthroscopy. In the case of different opinions between the two, the radiologists' diagnosis was considered more reliable.

All arthroscopies were performed by 2 experienced knee surgeons in a hospital environment with complete preoperative and postoperative care. A 4 mm Karl-Storz arthroscope with a 30-degree angle was used. Standard arthroscopic portals were used. ; the inferolateral portal for the arthroscope, and the inferomedial portal for the probe. Before any intervention, all knee interior structures were examined with the probe. Chondral defects were classified as positive if were more than 2^nd ^grade according to the Outerbridge classification [7] and measured more than 1 cm in diameter. A cruciate ligament was considered to be torn if it was completely disrupted at one of its attachments to bone or in its substance, or if laxity (partial tear) could be demonstrated with a probe. All arthroscopic findings were photographed and registered. For further evaluation all arthroscopic findings were considered accurate and served as reference base.

MRI diagnoses and clinical findings were placed into one of four categories after arthroscopic evaluation. A result was considered a true-positive if the clinical or MRI diagnosis was confirmed by arthroscopic evaluation. A result was considered a true-negative if the diagnosis of no tears was confirmed by arthroscopy. A result was considered a false-positive if the arthroscopy was negative but the results were positive at the clinical examination or on the MRI. If the arthroscopy was positive but the clinical examination and MRI were negative, this was considered a false-negative result.

Statistical analysis was used to calculate sensitivity, specificity, positive predictive value (PPV) and negative predictive value (NPV), in order to assess the realibility of the clinical and MRI results. 95% confidence intervals for sensitivity and specificity, as well as positive (LR+) and negative likelihood ratios (LR-) and areas under the ROC curve (AUC) were calculated.

## Results

**Figure 1:**
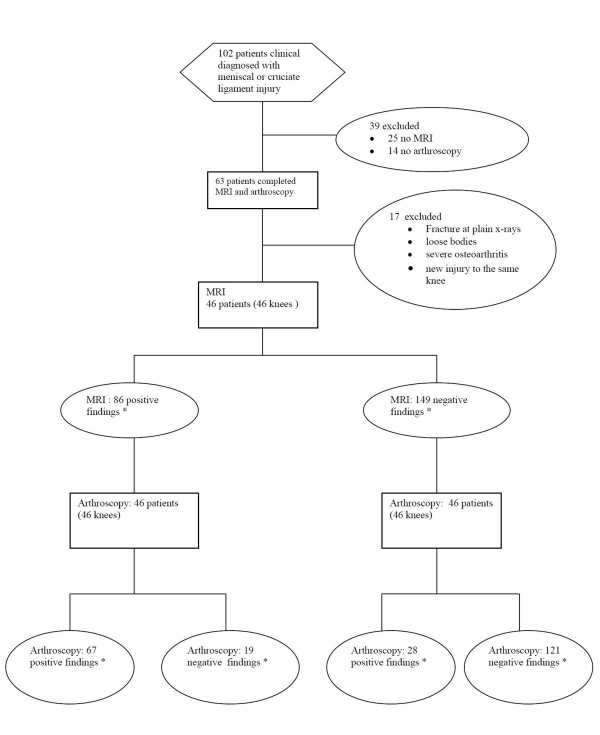
**Standards for Reporting of Diagnostic Accuracy flow diagram**. * Medial meniscus, lateral meniscus, Anetrior cruciate ligament, posterior cruciate ligament ruptures and chondral injuries.

One hundred two patients after clinical examination were diagnosed with meniscal or cruciate injury and underwent definitive treatment with arthroscopy, during the studied period. After the application of the exclusion criteria we were able to identify 46 patients (30 males) that were further examined with MRI preoperatively. The mean age was 32 (18 – 45) years. Right knee injury presented to 21 whereas left knee injury to 25 patients. Table 1 summarizes the patients' demographics. Arthroscopy revealed 33 medial meniscus tears, 21 lateral meniscus tears, 23 ACL injuries, 3 PCL injuries and 19 grade 2, 3 or 4 chondral defects (Table 2). The STARD patient flow diagram is shown in Figure 1.

**Table 1:** Demographic baseline data for the patients that fulfilled the inclusion criteria (N = 46)

***Demographic baseline data***	
Patients (N)	46
Males	30
Females	16
Side (Left/Right)	25/21
Mean Age (Range)	32 (18 – 45)
Mechanism of injury (N)	
*Sport injury*	16
*Non sport injury*	18
*No history of injury*	12
Mean delay from injury to MRI (weeks) (N = 34)	6.9 (0–58)
Mean delay from MRI to arthroscopy (weeks) (N = 46)	2.7 (0 – 12)

**Table 2:** Total number of arthroscopic findings in patients that had previously examined with MRI

**Arthoscopic findings**	
Medial meniscus tears	29
Lateral meniscus tears	21
ACL injuries	23
PCL injuries	3
Chondral defects	19

After the classification of the MRI diagnoses in true positive, false positive, true negative and false negative the accuracy, specificity, sensitivity, PPV, NPV, the LR+ and LR- and the AUC were calculated and are demonstrated in table 3. The sensitivity of MRI for medial meniscus rupture was 83% and the specificity 69%. The area under the ROC curve was 0.75. For ACL ruptures the percentage was higher with sensitivity reaching 83%, specificity 89% and area under the ROC 0.86 Slightly inferior were the results for lateral meniscus rupture with accuracy at 77%, sensitivity 62%, specificity 88% and AUC 0.75. Significantly inferior was the accuracy of MRI as far as the chondral defects concerns, with values of 60% in accuracy, 42% in sensitivity, 73% in specificity and AUC 0.57. We have separately evaluated the predictive value of clinical examination as far as the meniscal and anterior cruciate ligament injuries concern. Overall, clinical examination revealed 40 meniscus tears and 25 cruciate ligament injuries. Table 4 demonstrates results for the diagnostic value of the clinical examination obtained from this study.

**Table 3:** Results of the data analysis: Accuracy, sensitivity, specificity, positive predictive value (PPV), negative predictive value (NPV), positive likelihood ratio (LR+), negative likelihood ratio (LR-) and area under the ROC curve (AUC) of magnetic resonance imaging to evaluate lesions of the medial meniscus, lateral, meniscus, anterior cruciate ligament, posterior cruciate ligament, and articular cartilage.

	**Medial meniscus tears**	**Lateral meniscus tears**	**ACL injuries**	**PCL injuries**	**Chondral injuries**
**Accuracy**	81%	77%	86%	98%	60%
**Sensitivity (95% CI) **	83% (63 – 93)	62% (30 – 81)	83% (60 – 94)	100% (31 – 100)	42% (21 – 66)
**Specificity (95% CI)**	69% (41 – 88)	88% (68 – 97)	89% (70 – 97)	98% (80 – 97)	73% (52 – 88)
**PPV**	83%	81%	90%	75%	53%
**NPV**	69%	74%	86%	100%	63%
**LR+**	2.64	5.36	7.43	45	1.56
**LR-**	0.25	0.43	0.19	0	0.79
**AUC**	0.75	0.752	0.86	0.98	0.57

**Table 4:** Results of the data analysis for the clinical examination. Results were significantly inferior to MRI.

	**Medial meniscus tears**	**Lateral meniscus tears**	**ACL injuries**
**Accuracy**	60%	55%	72%
**Sensitivity (95% CI)**	65% (44 – 82)	30% (13 – 54)	68% (46 – 84)
**Specificity (95% CI)**	50% (26 – 73)	75% (53 – 89)	77% (54 – 91)
**PPV**	65%	50%	80%
**NPV**	50%	56%	68%
**LR+**	1.30	1.2	2.99
**LR-**	0.69	0.93	0.41
**AUC**	0.57	0.525	0.726

## Discussion

The purpose of this study was to demonstrate the diagnostic value of MRI in diagnosing the presence or absence of the most common injuries of the knee; the meniscus tears, the cruciate ligament ruptures and the chondral defects.

There are studies that support the view that the diagnostic accuracy of the MRI could affect in a critical way the treatment pathway of knee injuries. McKenzie et al [8] have studied 332 patients' diagnosis before and after MRI. The diagnosis was initially based upon the clinical examination and the therapeutic procedure was decided before MRI. 57 from 113 clinically positive before MRI meniscal tears were not confirmed with MRI. This result leaded to revaluation and differentiation of treatment in 62% of the patients. From those patients programmed for surgery only 38% finally underwent arthroscopy. In another study, Weinstabl et al [9] randomly distributed patients with positive meniscus rupture tests in two groups. All the patients of the first group had MRI examination before arthroscopy. In this group only 2% of patients didn't have positive findings during arthroscopy. Second group patients underwent arthroscopy, based only to the findings of the clinical examination. In this group, only in 30% of patients arthroscopy confirmed the findings of clinical examination.

However, in our study, MRI showed false results in significant proportion. For example as far as medial meniscus concerns there were 5 false positive and 5 false negative diagnosis whereas for lateral meniscus there were 8 false positive and 3 false negative diagnosis (PPV 83% and 81%, NPV 69% and 74% for medial and lateral meniscus tears respectively). As far as the chondral lesions concerns the MRI results were even more inferior with PPV and NPV reaching 53% and 63% respectively.

**Figure 2:**
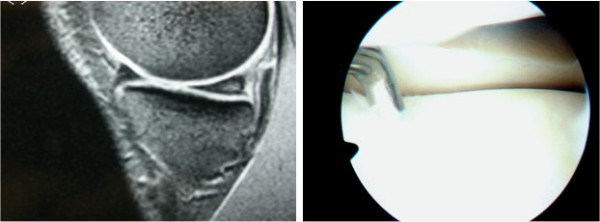
Left: Intrabody signal of the posterior aspect of meniscus, without extension to the articular surface. Right: At arthroscopy the meniscus appearance was normal.

There are several explanations for the misleading results of MRI regarding the menisci. Firstly, meniscal tears and meniscus degenerative changes have the same appearance in MRI, by giving high signal within the meniscus [10]. Diagnosis then depends on the expansion of the high signal line towards meniscus articular surface [11] (FIGURE 2). Moreover, one of the most frequent causes for false positive MRI regarding the lateral meniscus is the misinterpretation of the signal coming from the inferior knee artery [12]. Helman et al [13] accredited in this structure about 38% of false positive MRI results. Often, the popliteal bursa or Humphrys' ligament may mimic posterior lateral meniscus tears as well [14,13]. McKenzie et al [15] summarized the four most common reasons for false positive diagnosis; wrong diagnosis due to variable anatomic structures, overestimation of pathology countered as meniscus tear (for example chondral injuries that mimic meniscus tears), false negative arthroscopic findings and tears within the meniscus without expansion to the articular surface. On the other hand the false negative results seem to occur exclusively from misinterpretation of MRI [16,14,1].

**Figure 3:**
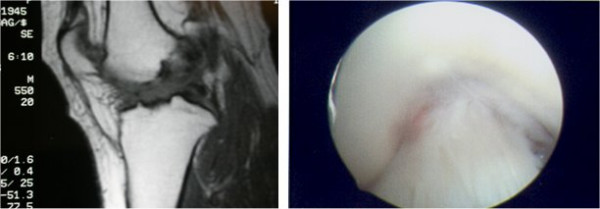
Left: Abnormal appearance of the ACL, suggesting a rupture. Right: At arthroscopy, the ACL appeared normal.

As far as the cruciate ligaments concerns, our study showed that from the 27 ACL ruptures diagnosed during arthroscopy 8 of them were missed by the MRI, leading to NPV of MRI for ACL ruptures of 86%. Causes of that target loss are easily recognized; firstly, in cases with ligament rupture without ligamentum mucosum rupture, MRI gives false negative results. Additionally, ruptures near ligaments' insertion may be missed and MRI examination reveals an intact ACL. On contrary, false positive ACL ruptures occur in cases of intrabody mucosal or eosinophilic degeneration of the ACL [17,18]. (FIGURE 3).

The posterior cruciate ligament can be examined very well with MRI. Bibliography refers accuracy in ruptures higher than 90% [19,20,1]. In our study we evaluated only 3 PCL ruptures and all were identified by MRI. At the same time, one false positive result occurred (accuracy 98%, sensitivity 100%, specificity 98%). Even though our results agree with the bibliography data, the number of cases is too small for statistical significant conclusions. However, surgeons must always bear in mind that PCL is difficult to investigate during arthroscopy because of its anatomic position, and many times there are arthroscopic false negative results.

**Figure 4:**
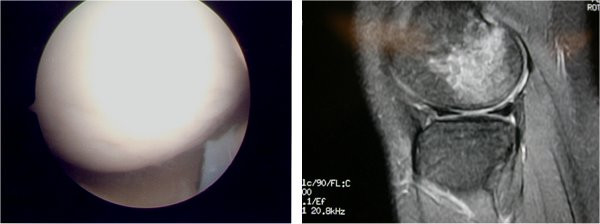
Right: MRI suggesting damage at the articular cartilage. Left: At arthroscopy, the cartilage appeared normal.

**Figure 5:**
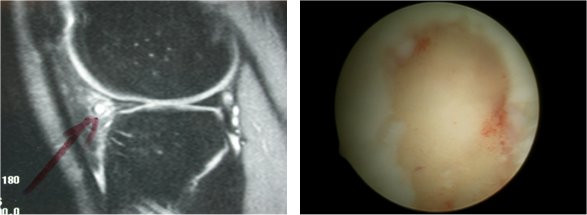
Left: Radiologist points out possible meniscal cyst. Right: At arthroscopy surgeon faced extensive articular cartilage damage.

In this study, from 19 grade 2, 3 or 4 chondral defects (diameter >1 cm) diagnosed arthroscopically only 8 were preoperatively described in MRI (PPV 53%) and additionally, there were 7 false positive diagnosis. In total accuracy was 60%, sensitivity only 42% and specificity 73% (FIGURES 4, 5). In many cases, subchondral bone bruises that are frequently described in MRI, are mistaken with chondral defects, leading to false positive results (FIGURE 4). They remain though important cause of pain and morbidity. Additionally, one must never forget that preoperative MRI mainly focuses on meniscal and cruciate ligament injuries. As a result, chondral lesions are often underestimated and misdiagnosed by MRI [21,22]. Postoperative new examination with MRI that focus on chondral defects leads to improvement of the diagnostic results [23,21,22].

Other authors however, like Heron et al [24], have shown that MRI can satisfactory reveal the 2^nd ^and 3^rd ^grade chondral defects as well as damages at the patellar articular cartilage, but is not accurate for smaller injuries like fibrilization or small fissuring in articular hyaline cartilage. Similar results were reported from Ochi et al [23] who showed that the sensitivity of MRI increased (from 40% to 71%) when MRI reading was done retrospectively, after the arthroscopic findings were registered. Especially, in chondral lesions with full thickness loss of cartilage and large-deep erosions the retrospectively calculated MRI sensitivity was 100% and 75% respectively. On the other hand site surface injuries, fibrillization or shallow small cuts were not well described, not even post-arthroscopically. Furthermore, according to Mori et al [22], usage of modern, improved techniques, can not only reveal the size of chondral lesions but to distinguish partial from full depth chondral damages as well.

There is no doubt that the radiologist's experience and training are very important factors in interpretation of MRI. At the same time reliable statistical data of the diagnostic value of MRI are also related with the independent base of reference. Regarding knee MRI, in most of the studies and in our study as well, the base of reference is arthroscopy. This presupposes that arthroscopy is 100% accurate and allows for the diagnosis of every possible knee pathology. This is not always the case [25,16] ; arthroscopy is a technical demanding procedure and the results are varying according to surgeons' experience, especially in difficult cases. From the 13 false positive results of our study, the majority referred to posterior meniscus tear. Nevertheless the belief is that, even in these cases, the meniscus pathology existed but failed to be discovered during arthroscopy [12,26]. Especially the inferior surface of posterior aspect of the medial meniscus is difficult to be reached with a probe and often rupture at that point can be missed. Nowadays, the overall accuracy of arthroscopy varies between 70–100%, depending on the surgeons' experience [16,27-29]. This fluctuation inevitably raises questions, regarding the reliability of the MRI results classification on true or false [30].

In the everyday practice, based on clinical examination that comes first, surgeons decide whether must proceed to further laboratory tests, MRI, conservative or surgical treatment. But how precise can clinical examination be? There seems to be disagreement regarding the answer to this question. Investigations support that the accuracy of clinical examination compared with arthroscopic findings ranges between 64–85% [31,32]. Rose et al [18] found that clinical examination is as accurate as MRI in diagnosing meniscal tears and ACL ruptures, so they concluded that MRI because of its high cost is not necessary in patients with clinical suspicion of meniscus and cruciate ligament tears. Similar conclusion was reported by Boden et al [33] who supported that when clinical examination sets the diagnosis of meniscus damage, MRI will not change treatment decisions.

On the other hand, Ruwe et at [34] reported that preoperative MRI can prevent unnecessary arthroscopy in 50% of the patients, so is of great value and must be done preoperatively. Boeree et al [35] believe that clinical examination is of minor significance with sensitivity in diagnosing medial meniscus, lateral meniscus and ACL tear of 67%, 48% and 55% respectively. Similar conclusions were reported by Jackson et al [36] who concluded that negative MRI for meniscus or cruciate ligament tears can discourage diagnostic arthroscopy even if clinical examination is positive for injury. The results of our study come in agreement with these studies, confirming a quite low diagnostic performance of the clinical examination (Table 4).

In summary, from our results, the accuracy of MRI in medial and lateral meniscus tear was 81% and 77% respectively, whilst for ACL and PCL rupture was 86% and 98% respectively. In the existing bibliography the accuracy of MRI reaches 90% in medial meniscus and ACL injuries, is lesser in lateral meniscus injury and slightly higher in PCL injuries [19,26,20,37,1,38]. Most of the studies agree that MRI has low accuracy and sensitivity as far as chondral defects concerns [12,23,39]. The same has been shown in the current study, with the accuracy to be only 60% and the sensitivity and specificity 42% and 73% respectively.

**Figure 6:**
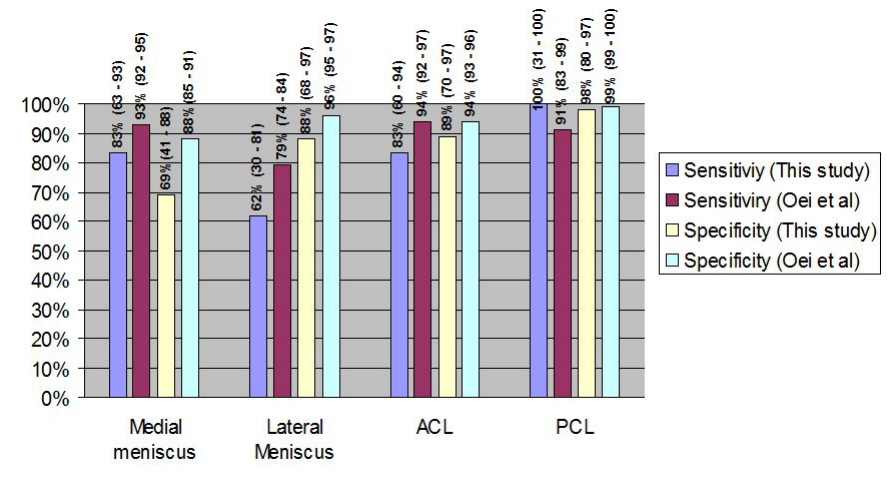
Comparison of the results (mean values and 95% confidence intervals) from this study compared with the results of the meta-analysis by Oei et al [40].

It is true that our results have yield worst diagnostic value of MRI in comparison with the results of larger multicenter studies [1] and of large systematic reviews [40] (FIGURE 6). This can be attributed to the limitations of the current study, which is a retrospective non randomized study with relatively small number of patients. Especially, the patients with a PCL injury were too few, in order to draw significant results. However, it is our believe that our findings mirror the reality that the average Orthopaedic surgeon will face during his everyday clinical practice.

## Conclusion

In conclusion, the present study supports that MRI is very helpful in diagnosing meniscal and cruciate ligament injuries. But in a countable percentage reports with false results and in chondral defects its importance is still vague. Nowadays patients' expectations are maximal and taking in account that MRI false or misleading results can be as high as 20% to 30% in specific knee pathologies it is concluded that arthroscopy still remains the gold standard in diagnosing the internal knee lesions. Undoubtedly new techniques and more powerful tomographers will improve MRI's accuracy leading to better diagnostic equipment in knee injuries.

In any case, what one must always have in mind is that diagnosis alone is no the end point of the treatment and does not solve the problem. It is the beginning of new thoughts and actions one must follow to achieve accurate prognosis and correct treatment. In order to plan and apply the correct treatment pathways, the most important is not statistics or cost effectiveness data. Clinical experience and adequacy of the surgeon always have the greatest value, when it comes to the assurance of the patient optimal treatment.

## Competing interests

The authors declare that they have no competing interests.

## Authors' contributions

VN, CS and EC were involved in collecting patient details, reviewing the literature, drafted and proof read the manuscript, PG and NE were involved in drafted and proof read the manuscript, SP was involved in the operation of patients and registered the clinical findings, GP is the senior author and was responsible for final proof reading of the article. All authors have read and approved the final manuscript.
